# Management of post lobectomy subcutaneous emphysema; a case report with literature review

**DOI:** 10.1016/j.amsu.2021.102610

**Published:** 2021-07-31

**Authors:** Razhan K. Ali, Fahmi H. Kakamad, Shalaw Hama ali Abdalla, Shakhawan I. Hussein, Abdulwahid M. Salih, Rawezh Q. Salih, Shvan H. Mohammed, Dahat A. Hussien, Marwan N. Hassan, Berwn A. Abdulla, Hiwa O. Abdullah, Snur Othman, Tomas M.Sharif M. Mikael

**Affiliations:** a Shar Hospital, College of Medicine, Sulaimani, Iraq; b University of Sulaimani, Sulaimani, Iraq; c Smart Health Tower, Madam Mittarand Street, Sulaimani Iraq; d Kscien Organization, Hamdi Street, Azadi Mall, Sulaimani, Iraq; e Hiwa Hematology and Oncology Hospital, Sulaimani, Iraq

**Keywords:** Lobectomy, Subcutaneous emphysema, Surgical emphysema, Negative pressure

## Abstract

**Introduction:**

Subcutaneous emphysema is an extremely rare complication after lobectomy. The current study aims to report a case of lung cancer developing extensive subcutaneous emphysema after lobectomy.

**Case presentation:**

A 73-year-old man presented with dyspnea and cough for one month duration associated with wheeze and sputum. He was a chronic heavy smoker (100 pack/year). Work up revealed squamous cell carcinoma. Although he had poor pulmonary function tests, he underwent left upper lobectomy. On the fifth postoperative day, he was discharged from the hospital as there was no air leak and the lung remained expanded 15 hours after clamping of the thoracostomy tube. Two days later, the patient developed generalized subcutaneous emphysema. The patient was re-admitted to the hospital and a thoracostomy tube was inserted. The lung expanded upon insertion while the subcutaneous emphysema remained the same and even slightly increased over night. A 3 cm incision was made at the left infra-clavicular area and a negative pressure applied to it. The subcutaneous emphysema completely subsided a few hours after this intervention.

**Discussion:**

Because of the benign course, the majority of cases of subcutaneous emphysema (mild to moderate) only need nonoperative management alongside treatment of the predisposing factors. These patients may need nothing other than bed rest, good analgesia, supplemental oxygen, and reassurance.

**Conclusion:**

Subcutaneous emphysema after lobectomy prolongs hospital stay. It mainly occurs in cases with poor pulmonary function tests, steroid use, and those with extensive adhesion.

## Highlights

• Subcutaneous emphysema is one of the complications following thoracic surgery.
• Subcutaneous emphysema might be a benign and self-limiting condition.
• It might be a serious condition that ends with respiratory failure and death.
• In this report, a case of lung cancer developing subcutaneous emphysema after lobectomy discussed.

## 1 Introduction

Post-operative complications after lobectomy are common ranging from 10 % to 50 %, which increases with advancing age [1]. The most frequent minor complications are atrial fibrillation and prolonged air leak, with some serious complications leading to respiratory failure and death [2]. The mortality rate for open lobectomy is slightly higher (3.13 %) than VATS lobectomy (1.19 %) [3]. Subcutaneous emphysema is one of the complications following thoracic surgery. It is caused by air leakage from a pulmonary fistula in almost all cases after operation [4]. However, subcutaneous emphysema might be a benign and self-limiting condition that usually responds to conservative management or a serious condition that ends with respiratory failure and death [5,6].

The current study aims to report a case of lung cancer developing subcutaneous emphysema after lobectomy. The report has been arranged in line with SCARE 2020 guidelines with a brief literature review [7].

### 1.1 Patient's information

A 73-year-old retired man presented with dyspnea and cough of one month duration associated with wheeze and sputum. He was a chronic heavy smoker (100 pack/year). The patient was a known case of chronic obstructive pulmonary disease (COPD). He was on prednisolone 10mgx2, and on a salbutamol inhaler. Past surgical and family history were unremarkable.

### 1.2 Clinical findings

An active man had bilateral diffuse wheeze with decrease air entry on the left upper zone. Vital signs were normal apart from low oxygen saturation (87–89 %) on room air.

### 1.3 Diagnostic assessment

Hematological investigations were within the normal range. The chest X-ray showed a left upper zone ill-defined opacity. Computed tomography (CT) scan demonstrated a left upper lobe mass measuring 5*6 cm with speculated outlines suggestive of bronchogenic carcinoma. Bronchoscopy revealed left upper lobe bronchial obliteration by a friable mass. Biopsy confirmed squamous cell carcinoma of the lung. Positron emission tomography (PET) scan was unremarkable for metastasis. Oncologically, the patient was a candidate for left upper lobectomy while medically he was deemed unfit (FEV1 0.9 L, 59 %).

### 1.4 Therapeutic intervention

The patient was referred to a pulmonologist for proper management of their COPD. After one month of medical therapy, which included a high dose of steroid, bronchodilators, and expectorant, the FEV1 increased to 1.25 L. Despite still not being considered medically fit, a multidisciplinary team decided on lobectomy, owing to the localized disease and curative potential in pursuing a more aggressive approach.

In right lateral position, through a classical posterolateral incision, left upper lobectomy was performed. Within the operation, the left pulmonary artery was injured inadvertently and repaired by a 6.0 suture material (Prolene). The patient had a delayed recovery from general anesthesia and admitted to the intensive care unit (ICU) for one night. The next day he was transferred to the ward.

**Fig. 1:**
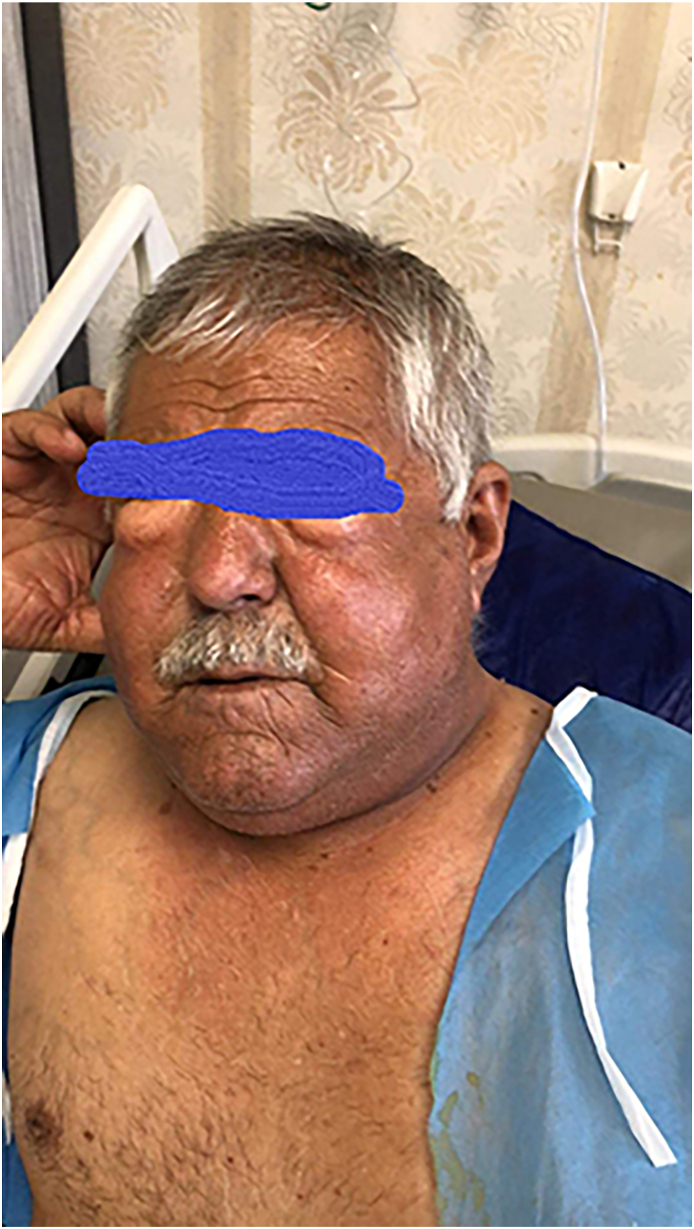
Photo of the patient seven days after lobectomy showing diffuse subcutaneous emphysema involving all of the body.

On the fifth postoperative day, he was discharged from hospital as there was no air leak and the lung remained expanded 15 hours after clamping of the thoracostomy tube. Two days later, the patient developed generalized extensive subcutaneous emphysema; so much so that it included his face and periorbital area. (Fig. 1). Chest x-ray showed a left side pneumothorax. CT scan did not show any track between the chest wall and pleura. The patient was re-admitted to the hospital and a thoracostomy tube was inserted; the lung expanded upon insertion while the subcutaneous emphysema remained the same and even slightly increased over night. A 3-cm incision was made at the left infra-clavicular area and a negative pressure applied to it. Fortunately, the subcutaneous emphysema completely subsided a few hours after this intervention.

### 1.5 Follow up

The patient remained healthy and without subcutaneous emphysema 30 days after the operation, he was referred to the oncology center for chemoradiotherapy.

## 2 Discussion

Subcutaneous emphysema is a condition caused by an accumulation of air inside the tissues under the skin. While it is more frequent in the soft tissue of the chest and neck, it can also occur in the soft tissue of other parts of the body [8]. It may spread rapidly and involve all over the face, chest, upper extremities, and lower extremities [9]. If it occurs secondary to a surgical procedure, it is called surgical emphysema [10]. In this case, air bubbles could be detected even in both lower limbs. There are a number of causes, including blunt or penetrating trauma, cancer, infectious process, iatrogenic, and also may occur spontaneously [8]. It can also occur following chest tube insertion, tracheal intubation, and upper gastrointestinal instrumentation; it may present in association with pneumothorax or pneumomediastinum [10].

Subcutaneous emphysema could be a cosmetical issue in some situations rather than a serious illness. The clinical presentation includes, swelling, dysphagia, dysphonia and pain. Visual problems may also occur due to periorbital swelling [11]. In the current case, the patient could not open his eyes. However, in some conditions the symptoms can be more extensive, worsening rapidly, disfiguring, and threatening to life which requires immediate intervention [12]. Srinivas and colleagues reported that surgical emphysema can be more extensive resulting in cutaneous tension, difficulty in swallowing, dysphonia, and pneumoperitoneum [13]. It is rarely reported that it may lead to respiratory failure and upper airway obstruction [6,9]. The risk factors for developing subcutaneous emphysema postoperatively include poor pulmonary function (DLCO less than 80 % predicted, presence of pleural adhesion, and steroid use in high dose for more than one month [14]. This patient had all of these risk factors.

The majority of cases of subcutaneous emphysema are easy to diagnose because of the specific clinical manifestations [8]. The proper way of diagnosis requires a detailed history and examination to assess the crepitation. The radiological examination can confirm the diagnosis by demonstrating the presence of air under the skin in the affected area [15]. Chest X-ray demonstrates a radiolucent striation of gas that outlines the fibers of the pectoralis major muscle [8]. However, the air in the soft tissue of the chest may obscure critically serious conditions such as pneumothorax [8]. Additional imaging as CT scan of the affected area is essential in patients who are stable [8,16]. It is rarely reported in the literature that subcutaneous emphysema has been mistaken with angioedema due to similarity in manifestation such as difficulty in breathing and fascial swelling [17]. However, one can differentiate between the two because emphysema spares the lips and crepitus on palpation is specific for subcutaneous emphysema [17].

Because of the benign course, the majority of cases of subcutaneous emphysema (mild to moderate) only need nonoperative management with the treatment of the predisposing factors [18]. These patients may need nothing other than bed rest, good analgesia, supplemental oxygen, and reassurance [17]. However, in more extensive conditions, it requires some invasive techniques. Endotracheal intubation should be performed without delay if the patient presented with stridor and respiratory distress [9]. The infraclavicular incision under local anesthesia was reported by Herlan et al. [19]. The benefits of this incision include a rapid recovery of the subcutaneous emphysema and improvement in the appearance of the patient. However, there are some complications of this incision including, occlusion of the incision by a clot, bleeding, insufficient drain positioning depth, and cosmetic appearance [8]. However the infraclavicular technique has been considered as the procedure of choice [13]. In the current case, infra-clavicular incision with negative pressure was very effective in the allowing the progressive emphysema to subside.

Bech and associates reported that the insertion of a subcutaneous catheter is a better than the previous techniques. It is much less likely to produce a scar, although there are significant problems such as infection and blockage of the catheter by blood [11]. Kelly et al. described that the drainage of subcutaneous emphysema may be done by using a modified large bore subcutaneous drain, which has an excellent cosmetic result [20]. A subcutaneous chest tube is another effective method but it is associated with more complications such as infection, technical error, and rarely perforation of the right atrium [21,22]. The patients with subcutaneous emphysema usually recovered after 2–3 days or completely after 5–10 days [15].

In conclusion, subcutaneous emphysema after lobectomy prolongs hospital stay. It mainly occurs in cases with poor pulmonary function tests, steroid use and those with extensive adhesion.

## Consent

Written informed consent was obtained from the patient for publication of this case report and accompanying images. A copy of the written consent is available for review by the Editor-in-Chief of this journal on request.

## 3 Provenance and peer review

Not commissioned, externally peer-reviewed.

## Sources of funding

None to be mentioned.

## Declaration of competing interest

The author(s) declared no potential conflicts of interest with respect to the research, authorship, and/or publication of this article.
